# S1P_4_ Regulates Passive Systemic Anaphylaxis in Mice but Is Dispensable for Canonical IgE-Mediated Responses in Mast Cells

**DOI:** 10.3390/ijms19051279

**Published:** 2018-04-25

**Authors:** Joseph M. Kulinski, Richard L. Proia, Elisabeth M. Larson, Dean D. Metcalfe, Ana Olivera

**Affiliations:** 1 Mast Cell Biology Section, Laboratory of Allergic Diseases, National Institute of Allergy and Infectious Diseases (NIAID), National Institutes of Health, Bethesda, MD 20892, USA; Joseph.kulinski@nih.gov (J.M.K.); eml244@cornell.edu (E.M.L.); dmetcalfe@niaid.nih.gov (D.D.M.); 2 Genetics of Development and Disease Branch, National Institute of Diabetes and Digestive and Kidney Diseases (NIDDK), National Institutes of Health, Bethesda, MD 20892, USA; richardp@intra.niddk.nih.gov

**Keywords:** S1P_4_, *S1pr4*, sphingosine-1-phosphate, mast cell, anaphylaxis, chemotaxis, mediator release, degranulation, IL-33

## Abstract

Mast cells are key players in the development of inflammatory allergic reactions. Cross-linking of the high-affinity receptor for IgE (FcεRI) on mast cells leads to the generation and secretion of the sphingolipid mediator, sphingosine-1-phosphate (S1P) which is able, in turn, to transactivate its receptors on mast cells. Previous reports have identified the expression of two of the five receptors for S1P on mast cells, S1P_1_ and S1P_2_, with functions in FcεRI-mediated chemotaxis and degranulation, respectively. Here, we show that cultured mouse mast cells also express abundant message for S1P_4_. Genetic deletion of *S1pr4* did not affect the differentiation of bone marrow progenitors into mast cells or the proliferation of mast cells in culture. A comprehensive characterization of IgE-mediated responses in S1P_4_-deficient bone marrow-derived and peritoneal mouse mast cells indicated that this receptor is dispensable for mast cell degranulation, cytokine/chemokine production and FcεRI-mediated chemotaxis in vitro. However, interleukin-33 (IL-33)-mediated enhancement of IgE-induced degranulation was reduced in S1P_4_-deficient peritoneal mast cells, revealing a potential negative regulatory role for S1P_4_ in an IL-33-rich environment. Surprisingly, genetic deletion of *S1pr4* resulted in exacerbation of passive systemic anaphylaxis to IgE/anti-IgE in mice, a phenotype likely related to mast cell-extrinsic influences, such as the high circulating levels of IgE in these mice which increases FcεRI expression and consequently the extent of the response to FcεRI engagement. Thus, we provide evidence that S1P_4_ modulates anaphylaxis in an unexpected manner that does not involve regulation of mast cell responsiveness to IgE stimulation.

## 1. Introduction

Sphingosine-1-phosphate (S1P) is a sphingolipid mediator that critically regulates multiple cellular processes including proliferation, survival, chemotaxis and immune regulation. S1P elicits these functions by binding five known G-protein coupled receptors (GPCRs), designated as S1P_1–5_, or by acting on its intracellular targets [1]. Unlike S1P_1–3_ receptors which are expressed ubiquitously, S1P_4_ exhibits preferential expression in lymphoid and hematopoietic organs and cells [2]. S1P_4_ has been reported to regulate neutrophil counts in circulation [3] and trafficking [4,5], dendritic cell function [6] and to modulate certain lymphocyte functions [6,7,8,9]. Global genetic deletion of S1P_4_ in mice results in elevated serum IgE levels, enhanced T helper 2 (Th2)-and Th17-dominated immune responses and diminished Th1-responses [6]. However, the role of S1P_4_ in immune cells is still not well understood.

Mast cells are tissue-resident cells commonly associated with Th2 immediate hypersensitivity reactions. Mast cells recognize IgE-bound antigen (Ag) through the high-affinity receptor for IgE (FcεRI) expressed at the plasma membrane. Aggregation of FcεRI by IgE/Ag initiates signaling cascades leading to the release of both early and late mediators that cause immediate allergic reactions and contribute to chronic inflammation [10,11]. In addition to FcεRI, mast cells express other cell surface receptors that allow mast cells to respond to signals in the microenvironment that modulate FcεRI-mediated responses [12,13]. These signals and their receptors may be significant factors in the susceptibility to, or severity of, anaphylaxis in allergic individuals [14]. 

Elevated S1P in inflamed tissues or produced endogenously by mast cells is considered one of the factors regulating FcεRI-induced responses [15,16]. Previous studies in mast cells have indicated that following FcεRI-mediated activation, S1P is generated and induces ligand-dependent “transactivation” of S1P_1_ and S1P_2_ receptors expressed on these cells [17]. S1P_1_ transactivation is reported to mediate the migration of mast cells toward Ag [17,18]. S1P_2_ enhances FcεRI-induced degranulation, although its contributions to degranulation may depend on the type of mast cell studied and culture conditions used [17,18,19].

Here, we show that mouse mast cells express S1P_4_ receptor in addition to S1P_1_ and S1P_2_. Based on the Th2-skewed phenotype of mice lacking S1P_4_ and the role of S1P in regulating mast cell responses, we sought to better understand the role of S1P_4_ in FcεRI-mediated stimulation and allergic responses. As it will be shown, global genetic deletion of *S1pr4* resulted in exacerbation of IgE-mediated systemic anaphylaxis, although S1P_4_ was dispensable for normal FcεRI-mediated activation in *S1pr4*-deficient cultured mast cells. Our data suggest that the enhanced anaphylaxis in mice lacking S1P_4_ is not directly linked to intrinsic alterations in mast cells, but instead may be secondary to the higher levels of IgE in vivo, which would promote membrane expression of FcεRI and thus a stronger stimulus. Our experiments also revealed an unexpected role for S1P_4_ in the negative regulation of innate mast cell degranulation in response to co-stimulation with IgE/Ag and IL-33 [20].

## 2. Results

### 2.1. S1P_4_ Is Expressed in Mast Cells

**Figure 1:**
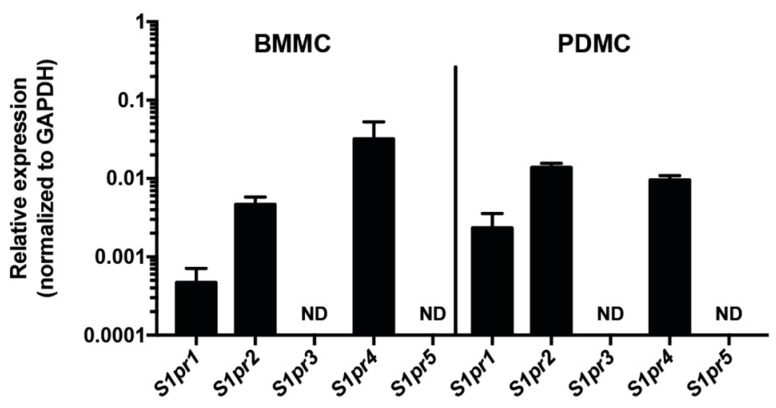
S1P_4_ is expressed in mast cells. Quantitative PCR data showing the relative expression of S1P receptor message normalized to glyceraldehyde 3-dehydrogenase (GAPDH) in bone marrow-derived mast cells (BMMC, **left**) and peritoneal-derived mast cells (PDMC, **right**). Plots represent the mean ± SE (or SD for PDMC) of data pooled from 7 independent BMMC or 2 PDMC cultures. ND: Not detected.

Mast cells express mRNA coding for S1P_1_ and S1P_2_*,* receptors known to contribute to FcεRI-mediated mast cell responses [16,17]. We found that, in addition to the expression of *S1pr1* and *S1pr2*, mouse mast cells contained mRNA for S1P_4_ (but not S1P_3_ or S1P_5_) at comparable or higher relative levels than for S1P_2_ (Figure 1). Since S1P_4_ may play redundant, antagonistic or unique roles in mast cells compared to the other S1P receptors, we reasoned it was possible that deletion of this receptor could affect the expression of the other S1P receptors, which could in turn skew the functional outcome. However, the mRNA expression of the other S1P receptors in mast cells was not altered by *S1pr4* deficiency (Figure S1A, open bars). As the role of S1P_4_ in mast cells has not been examined, we next sought to characterize the growth of mouse mast cells obtained from *S1pr4*-deficient mice.

### 2.2. Maturation and Proliferation of S1pr4-Deficient Mast Cells In Vitro

Bone marrow-derived mast cells (BMMC) from *S1pr4*-deficient mice cultured in the presence of IL-3 and stem cell factor (SCF) differentiated with comparable kinetics to *S1pr4*^+/+^ BMMC, as evidenced by the increasing appearance over time in culture of a population of FcεRI and CD117 (KIT) double-positive cells, the characteristic cell surface markers of mast cells (Figure S1B top panel, and C). In addition, the absence of S1P_4_ had no significant effect on the total numbers of mast cells in culture (Figure S1B, bottom panel). Similar to BMMC, the growth and expansion of *S1pr4*-deficient mast cells obtained from peritoneal exudates (peritoneal-derived mast cells or PDMC), which are considered more mature than BMMC [21,22,23] and with functional characteristics of innate mast cells [24], was no different from *S1pr4*^+/+^ cells (Figure S1D). These data indicate that S1P_4_ receptor expression is not required for either the expansion of mature mast cells from the peritoneum or the in vitro differentiation/expansion of mast cells from bone marrow precursors.

### 2.3. Degranulation, Cytokine and Chemokine Responses in S1pr4-Deficient Mast Cells In Vitro 

**Figure 2:**
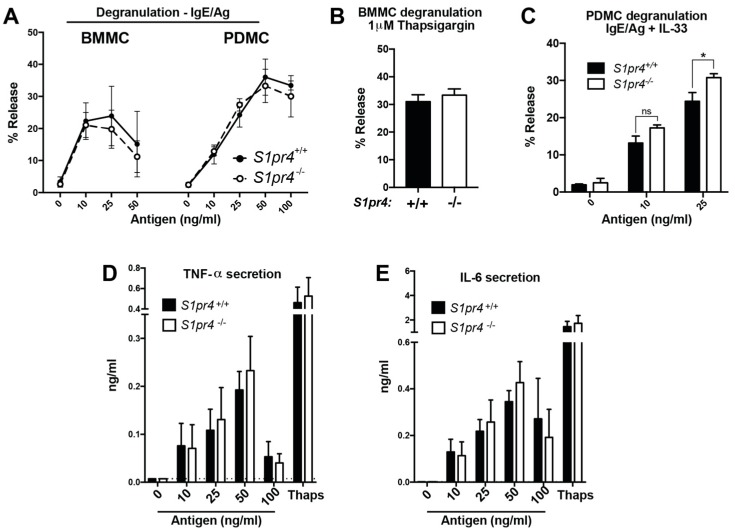

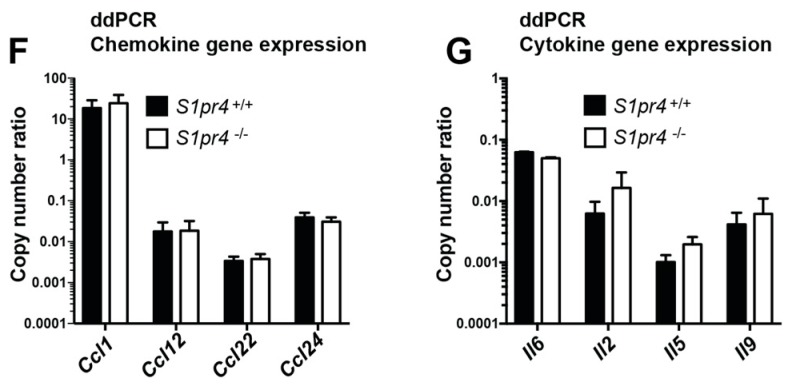
Analysis of degranulation, cytokine and chemokine responses in primary S1P_4_-deficient mast cells. (**A**–**C**) Degranulation response to antigen stimulation (**A**,**B**) or to pharmacological stimulation with thapsigargin (**B**). Mast cells from *S1pr4*^+/+^ (solid) and *S1pr4*^−/−^ mice (open) were grown in the presence of stem cell factor (SCF) and recombinant mIL-3 for 6–7 weeks (BMMC) or 14 days (PDMC) and sensitized with 100 ng/mL anti-dinitrophenyl (DNP)-IgE in cytokine-free media for 14 h. Degranulation was assessed by measuring the release of β-hexosaminidase into the media after 30 min of stimulation with the indicated concentrations of DNP (antigen; Ag) (**A**), 1 µM thapsigargin (**B**), or antigen in addition to 1 ng/mL recombinant IL-33 (**C**). Data represent the mean ± SE of results pooled from 4–8 independent cultures. (**D**,**E**) BMMC from *S1pr4^+/+^* (solid bars) and *S1pr4^−/−^* mice (open bars) were sensitized overnight with 100 ng/mL anti-DNP IgE in cytokine-free media. Cells were washed, stimulated with the indicated concentrations of Ag and the amounts of IL-6 (**D**) and TNF-α (**E**) secreted into the media measured by ELISA at 4 h post-stimulation. The limit of detection for IL-6 and TNF-α quantitation by ELISA are shown by a dotted line in panels C and D at 0.0018 ng/mL and 0.00721 ng/mL, respectively. Data is pooled from 4 independent cultures. (**F**,**G**) Validation by ddPCR of the normalized relative expression of select chemokines (**F**) and cytokines (**G**) identified as being variably upregulated in *S1pr4*^+/+^ and *S1pr4*^−/−^ BMMC cultures following Ag stimulation. Relative expression of *Il6* is included for comparison. Data show mean ± SE of values obtained from at least seven independent cultures of BMMC for each genotype. All comparisons between *S1pr4^+/+^* and *S1pr4^−/−^* cells were found to be not statistically significant unless otherwise indicated. * *p* < 0.05.

We next tested whether S1P_4_ might modulate degranulation in response to FcεRI stimulation in BMMC and PDMC cultures. Our analysis showed that *S1pr4*-deficient cultures bound comparable levels of IgE on the cell surface (Figure S2A–C) and showed similar degranulation to that of *S1pr4*^+/+^ cultures in response to all concentrations of Ag tested (Figure 2A). *S1pr4*-deficient and *S1pr4*^+/+^ cells also exhibited identical responses to pharmacological stimulation by thapsigargin, an inhibitor of Ca^2+^ uptake into the ER that causes increased cytosolic Ca^2+^ accumulation (Figure 2B). Further, degranulation in response to IgE/Ag was also similar in both groups of cultures in the presence of SCF (45.094 ± 0.862% in *S1pr4*^+/+^ and 42.443 ± 0.804% in *S1pr4*^−/−^ stimulated with 25 ng/mL Ag + 20 ng/mL SCF), which is known to synergize with FcεRI-mediated responses [14,25].

Cultured PDMC degranulate in response to a diverse group of cationic compounds, referred to as “mast cell secretagogues” such as substance P and compound 48/80, through a class of GPCRs known as Mas-related gene (Mrg) receptors expressed on these cells [24,26,27]. Degranulation of *S1pr4*-deficient PDMC in response to 5 to 50 µg/mL of compound 48/80 was indistinguishable from *S1pr4*^+/+^ mast cells (27.356 ± 8.997% in *S1pr4*^+/+^ and 20.334 ± 4.831% in *S1pr4*^−/−^ stimulated with 10 µg/mL compound 48/80). In contrast, FcεRI-induced degranulation in the presence of IL-33, a cytokine that orchestrates a variety of allergic inflammatory conditions through innate immune cells [20,28] and potentiates FcεRI mediated mast cell responses [25,29], was further potentiated in *S1pr4*-deficient PDMC compared to *S1pr4*^+/+^ (Figure 2C).

These effects were not observed in BMMC (15 ± 2% in *S1pr4*^+/+^ and 17 ± 2% in *S1pr4*^−/−^ stimulated with 25 ng/mL Ag and 1 ng/mL IL-33). Overall, the data indicates that S1P_4_ is dispensable for degranulation initiated through either FcεRI or the Mas-related GPCRs but diminishes the potentiating effects of IL-33 on FcεRI-mediated responses in innate mast cells.

Mast cells also generate a variety of cytokines and chemokines following activation of FcεRI as a result of enhanced gene expression, with IL-6 and TNF-α representing two of the most abundant and best characterized cytokines produced by BMMC [21,30,31]. *S1pr4* deficiency did not significantly alter FcεRI-induced transcription of IL-6 and TNF-α (relative *Il6* expression was 0.05609 ± 0.01661% in *S1pr4*^+/+^ and 0.0493 ± 0.01077% in *S1pr4*^−/−^ stimulated with 25 ng/mL) or their release into the media (Figure 2D,E) at any of the concentrations of Ag tested. To determine whether transactivation of S1P_4_ might regulate the expression of other cytokines or chemokines induced by FcεRI stimulation, as has been reported for S1P_2_, we performed a qPCR array to examine the relative expression levels of 84 key cytokines and chemokines with critical roles in various immune responses. From those whose expression was highest and/or induced by FcεRI activation, three cytokines (IL-2, IL-5 and IL-9) and three chemokines (Ccl12, Ccl22, Ccl24) exhibited relative expression levels that were on average ≥2-fold higher in stimulated *S1pr4*^−/−^ BMMC than in stimulated *S1pr4*^+/+^ controls (Table S1). The relative expression levels for several of the genes were highly variable between replicates probably due to the low abundance of these transcripts (100–1000 fold less abundant than those for IL-6) and the same was true, in general, for the fold change in expression between stimulated *S1pr4*^−/−^ and *S1pr4*^+/+^ cells in separate cultures and thus, to confirm the measurements on these cytokines/chemokines, we employed droplet digital PCR (ddPCR) technology for enhanced sensitivity and reproducibility [32]. We also determined IL-6 expression by ddPCR for comparison, as a negative control, and Ccl1 since this was one of the most highly upregulated messages in both *S1pr4*^−/−^ and *S1pr4*^+/+^ after stimulation and the average fold increase in *S1pr4*^−/−^ compared to *S1pr4*^+/+^ cells was nearly 2 fold (Table S1). Using ddPCR we were able to accurately quantify those low and high abundance transcripts to definitively conclude that activated *S1pr4*^−/−^ BMMC have indistinguishable responses to those of *S1pr4*^+/+^ cells in terms of cytokine/chemokine mRNA expression (Figure 2F,G). Only IL-2 and IL-5 showed a trend towards higher expression in stimulated *S1pr4*^−/−^ although this was not statistically significant. Altogether our data demonstrates no role for S1P_4_ expression in the normal functioning of FcεRI-mediated responses in vitro.

### 2.4. Regulation of Mast Cell Chemotaxis by S1P_4_

**Figure 3:**
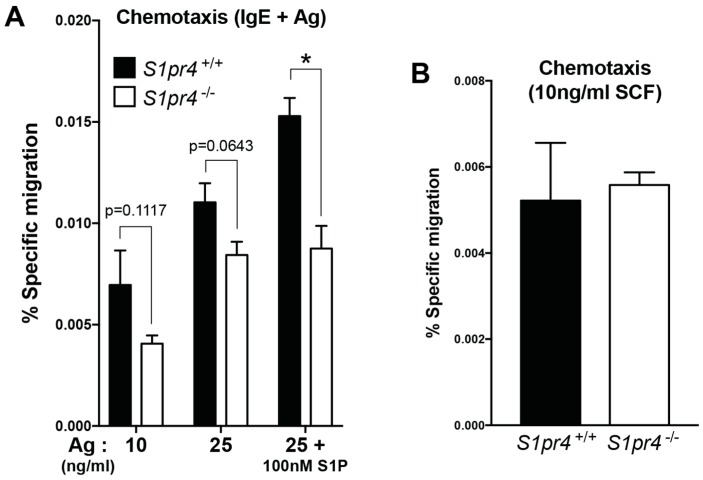
BMMC chemotaxis in the absence of *S1pr4* expression. (**A**) BMMC from *S1pr4*^+/+^ (solid bars) and *S1pr4*^−/−^ mice (open bars) grown in the presence of SCF and IL-3 for 6 weeks were sensitized with anti-DNP IgE in serum-free, cytokine-free media supplemented with 0.04% fatty acid-free bovine serum albumin (BSA) for 14 h. Sensitized cells were subject to transwell migration analysis using antigen (DNP; Ag) with or without 100 nM S1P as a chemoattractant at the indicated concentrations in the bottom chamber. (**B**) Chemotaxis of BMMC towards 10 ng/mL SCF in the bottom chamber. After 4 h incubation at 37 °C in 5% CO_2_, cells that migrated into the lower chamber were collected and counted. In each experiment, three to six technical replicates were performed for the *S1pr4*^+/+^ and *S1pr4*^−/−^ cultures. Percent specific migration was calculated by taking the average number of cells in the bottom chamber/total input cells × 100. Data represents the mean ± SE normalized migration for three independent BMMC cultures for each genotype. In each experiment, migration of *S1pr4*^+/+^ and *S1pr4*^−/−^ cultures were normalized to the average migration to Ag (10 ng/mL) across the 3 *S1pr4*^+/+^ cultures. * *p* < 0.05.

Various S1P receptors modulate chemotaxis in a variety of cell types [33]. In mast cells, S1P_1_ mediates migration toward Ag while overexpression of S1P_2_ appears to antagonize this process [17,18]. Transwell migration of BMMC towards Ag (Figure 3A) or towards SCF (Figure 3B) were minimally affected by the absence of S1P_4_ expression. Addition of 100 nM S1P did enhance the number of *S1pr4*^+/+^ BMMCs exhibiting specific migration towards Ag but had no effect on *S1p4*-deficient BMMC (Figure 3A). Thus, under these specific conditions, there was a trend towards reduced chemotaxis toward Ag in *S1p4*-deficient BMMC. However, the data suggest that the role of S1P_4_ in chemotactic mast cell migration is at best marginal.

### 2.5. Systemic Anaphylaxis in S1pr4^−/−^ Mice

**Figure 4:**
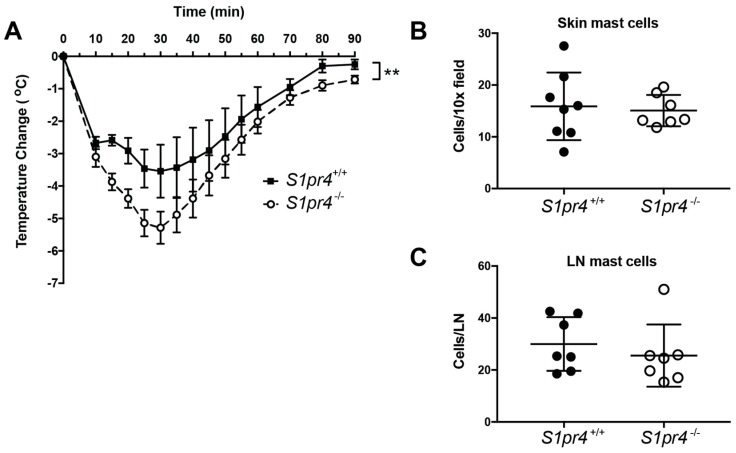
*S1pr4* deletion exacerbates PSA. (**A**) *S1pr4^+/+^* and *S1pr4^−/−^* mice were injected i.v. with 3 µg of mouse IgE. 24 h later, systemic anaphylaxis was induced by i.v. injection of 9 µg of anti-mouse IgE. Body temperature was monitored at the indicated times (S1pr4^+/+^ *n* = 4, S1pr4*^−^*^/*−*^ *n* = 7). The asterisks between the curves indicate significant differences (*p* < 0.001) between genotypes using a two way-ANOVA test. (**B**,**C**) Dorsal skin biopsies (**B**) and inguinal lymph nodes (LN) (**C**) harvested from *S1pr4^+/+^* and *S1pr4^−/−^* mice were fixed in 10% neutral buffer formalin, embedded in paraffin and sectioned. Three sections per skin biopsy and two sections per lymph node were stained with toluidine blue and eosin. Each dot represents the average number of metachromatic staining cells/10× field (**B**) or inguinal LN section (**C**) in one mouse and was calculated from five fields for each section examined, averaging values from 3 (**B**) or 2 (**C**) different sections for each tissue/animal. Floating bars represent the mean ± SE for each group of mice.

Mast cells grown and differentiated in the presence of IL-3 and SCF in culture may react differently to antigenic stimulation than cells undergoing activation during immune responses in vivo. To assess mast cell responses in *S1pr4*^−/−^ mice in vivo, we induced an anaphylactic response using a model of passive systemic anaphylaxis (PSA). *S1pr4*^−/−^ and *S1pr4*^+/+^ mice were sensitized with 3 µg of IgE to saturate IgE receptors prior to challenge with anti-IgE. Crosslinking of FcεRI on mast cells in this manner results in anaphylaxis, which is manifested in mice by a drop in body temperature. *S1pr4*^−/−^ mice exhibited increased hypothermia compared with *S1pr4*^+/+^ controls that was most apparent early on and was maintained throughout the course of induced anaphylaxis (Figure 4A). Histamine is a key vascular mediator released by mast cells that elicits anaphylactic symptoms in mice [34]. Previous reports indicate that anaphylactic reactions in *S1pr4*^−/−^ mice are indistinguishable from those in *S1pr4*^+/+^ mice when anaphylaxis is induced by systemic administration of exogenous histamine [35], suggesting that the differences observed in absence of S1P_4_ in this study following administration of IgE/anti-IgE are likely not due to an overall change in sensitivity to histamine. In addition, exacerbated anaphylaxis in *S1pr4*^−/−^ mice was unlikely due to a higher mast cell burden since the number of metachromatic mast cells present in toluidine blue stained tissues is similar to that observed in *S1pr4*^+/+^ controls (Figure 4B,C).

Since *S1pr4*^−/−^ mice exhibit elevated levels of circulating IgE ([6] and Figure S2D) and IgE is known to regulate the amount of mast cell surface FcεRI, which in turn can determine the extent of mast cell responses [36], we measured whether the expression of FcεRI on mast cells in peritoneal exudates from *S1pr4*^−/−^ mice was altered. Staining with a mAb specific for FcεRI (MAR-1) along with anti-IgE to measure signal from both occupied and unoccupied FcεRI [37] suggested that peritoneal mast cells ex vivo from unchallenged *S1pr4*^−/−^ mice express higher levels of FcεRI at the plasma membrane compared to *S1pr4*^+/+^ control cells (Figure S2E,F). Since S1P_4_-deficient PDMC or BMMC, once removed from the influence of higher IgE levels, show no differences in FcεRI expression (Figure S2A–C) or intrinsic alterations in their responses (Figure 2), it is reasonable to surmise that the exacerbated anaphylactic responses in *S1pr4*^−/−^ mice could partly be attributed to an increase in the expression levels of FcεRI due to an exposure to relatively high IgE levels.

## 3. Discussion

The importance and complexity of S1P signaling during allergic immune responses continues to emerge as we gain a greater understanding of how the various S1P receptors influence immune regulation. Mice deficient in S1P_4_ exhibit an allergy-prone phenotype [6], although little is known regarding the contribution of mast cells or other cell types to this condition. S1P generated following FcεRI activation in mast cells induces ligand-dependent transactivation of S1P_1_ and S1P_2_ which contribute to specific IgE-mediated responses [17]. In this study, we show that the S1P_4_ receptor is expressed in murine mast cells and that genetic deletion of *S1pr4* results in increased IgE-mediated anaphylaxis in mice. However, we find that the absence of S1P_4_ in the mast cell compartment does not cause alterations in IgE-mediated degranulation or cytokine/chemokine responses in vitro, and thus the increased anaphylactic responses seem to relate to mast cell-extrinsic influences in the *S1pr4* deficient environment surrounding mast cells in vivo. Although S1P_4_ was dispensable for IgE-mediated signaling under standard culture conditions, in the context of IL-33 co-stimulation, IgE-mediated degranulation was negatively modulated by S1P_4_, a finding of relevance given the involvement of the IL-33-mast cell axis in allergic inflammation [20,38,39].

Previous reports have implicated S1P receptors, particularly S1P_1_, in the regulation of mast cell chemotaxis towards Ag [17,18]. This process is likely to be integral to allergic conditions such as bronchial asthma and allergic rhinitis where mast cell accumulation in tissues is critical for the development of disease [40]. Inhibition of S1P production by mast cells [17,19,41], inhibition of S1P transport from mast cells to the extracellular medium [18], or knockdown of S1P_1_ [17], results in inhibition of mast cell chemotaxis towards Ag in vitro, supporting the concept that FcεRI triggering promotes mast cells migration via S1P generation, export and transactivation of the S1P_1_ receptor. Given that inhibition of Gi signaling, which functions downstream of both S1P_1_ and S1P_4_ [42], effectively blocks migration of mast cells towards Ag [17] and that signaling through both S1P_1_ and S1P_4_ receptors can affect actin dynamics through activation of the small GTPases Rac and Rho, respectively [43,44,45,46,47,48], a contributory role for S1P_4_ on mast cell chemotaxis might be expected. Even though S1P_4_ activates pathways involved in cell motility, it did not appear to have a relevant role in FcεRI-induced chemotaxis. Nevertheless, there was a trend towards reduced motility to Ag, and addition of S1P as a chemoattractant together with Ag promoted migration in *S1pr4*^+/+^ but not in *S1pr4*^−/−^ BMMC and thus a minor role for S1P_4_ in mast cell chemotaxis is possible. In general, this would be in agreement with reports that examine other immune cell types and implicate S1P_4_ in a contributory, albeit less prominent chemotactic role alongside S1P_1_ [9,49,50].

Our studies did not support a role for S1P_4_ receptors in regulating effector responses of Ag-stimulated PDMC or BMMC in our normal culturing conditions (in the presence of SCF and IL-3). Of note, under similar conditions, S1P_2_ did not affect IgE-mediated responses in PDMC or BMMC [51], although when cultured in the presence of IL-3 alone, a contribution for S1P_2_ in FcεRI-induced responses was manifested [51], a role which was also reported in human mast cells [17,51,52]. It is considered that BMMC cultured in the presence of IL-3 without SCF may present a more mucosal-like phenotype [53] than mast cells cultured in IL-3 and SCF. This distinction is, however, tenuous and it is unclear what culture conditions would better represent the phenotype of mast cells resident in tissues. Given the critical role of SCF in the location of mast cells within tissues and for mast cell maturation [54,55,56], experimental conditions that include SCF should be preferable and thus, our findings argue against a significant role for S1P_4_ in regulating mast cell function under homeostatic conditions. We cannot exclude, however, the possibility that signaling elicited through S1P_4_ in combination with other environmental cues under normal or pathological conditions [57,58] could modulate mast cell responses. In fact, our findings indicated that S1P_4_ negatively modulates the synergistic effect of IL-33 on IgE-mediated degranulation. These findings deserve further investigation since IL-33 is emerging as a critical player orchestrating allergic inflammation through innate immune cells, including mast cells. Increases in IL-33 in the epithelia are caused by barrier defects, microbiome alterations, irritants, allergens and other substances [28]. In addition to its effects on mast cell cytokine production [25,29], IL-33 promotes mast cell degranulation-associated responses leading to exacerbated sensitization to food or airway allergens [38,39]. Further, proteases released by mast cells during allergic reactions enhance the inflammatory potential of IL-33 by cleaving IL-33 into more active fragments [59], constituting a positive feedback loop for inflammation. As mast cells can also downregulate IL-33 actions in other models of inflammation [20], a better understanding of the mechanisms and circumstances under which S1P_4_ modulates IL-33 actions in mast cells may be beneficial for learning how to tamper certain allergic conditions.

We show here that mast cell-dependent, FcεRI-mediated anaphylaxis is more severe in *S1pr4^−/−^* mice, particularly at early phases of the response. Similarly enhanced anaphylactic responses to IgE/Ag in *S1pr2*^−/−^ mice were attributed to an impairment in the regulation of vascular tone during anaphylaxis in these mice and thus defective histamine clearance and recovery from anaphylaxis [35,51]. Indeed, *S1pr2*^−/−^ mice also have more severe anaphylaxis in response to vascular mediators such as histamine and PAF [35,51,60], which are released from mast cells and mediate vascular and temperature changes associated with IgE-induced anaphylaxis. In contrast, anaphylaxis induced by histamine administration in *S1pr4*^−*/*−^ mice was no different than that observed in *S1pr4*^+/+^ mice [35], suggesting that there are no significant alterations in the response to vascular mediators or in the recovery from anaphylaxis and implicating instead enhanced mast cell responses. The increased severity of anaphylaxis in *S1pr4*^−*/*−^ mice was unexpected given that cultured mast cells lacking S1P_4_ had no significant intrinsic alterations in their IgE-mediated responses in vitro. The heightened anaphylactic responses might be in part attributed to elevated levels of circulating IgE in these mice ([6] and Figure S2D), since IgE levels are known to regulate cell surface FcεRI expression and mast cell responsiveness [36]. In support of this notion, peritoneal mast cells from *S1pr4*^−*/*−^ mice expressed higher FcεRI levels than *S1pr2*^+/+^ controls ex vivo, unlike cultured *S1pr4*^−*/*−^ mast cells in vitro that lack chronic exposure to IgE. However, we cannot exclude the possibility that complex and dynamic cues in the tissues of *S1pr4*^−*/*−^ mice mold the phenotype of mast cells in a manner that cannot be recapitulated in vitro. There is also precedence for the notion that S1P_4_ may be indirectly influencing FcεRI-mediated mast cell degranulation in vivo through its documented function in other cell types [6,47,61].

In summary, our studies demonstrate that S1P_4_ is expressed in mast cells, but mast cell-intrinsic expression is dispensable for most IgE-mediated responses in vitro. We, however, unveil a modulatory role for S1P_4_ in the exacerbation of innate type mast cell degranulation by IL-33. This observation may be of importance and requires further study, especially in the context of allergic inflammation where IL-33 is key. S1P receptor expression and signaling is clearly important for allergic mast cell-mediated responses in vivo and studies employing mice that harbor conditional or tissue-specific *S1pr4* deletion will be critical for further dissecting the role of this molecule and its biology in the context of complex immunologic responses.

## 4. Materials and Methods 

### 4.1. Mice

Mice were maintained and used in accordance with NIH guidelines and animal study proposals approved by the NIAID (LAD2E; 1/12/2017) and NIDDK (K007-GDDB-15; 17/2/2015) animal care and use committee. *S1pr4^−/−^* mice and *S1pr4^+/+^* mice (referred to as WT mice) were obtained from crossing heterozygous mating pairs (strain B6.129P2-*S1pr4^tm1Dgen^*/J) from the Jacksons Laboratory (Bar Harbor, Maine). Mice had been backcrossed to C57/BL6 at least 7 times and maintained at NIH vivaria. Genotyping was performed using the following primers: (5′-GGC CTA CGT GGT CAA CGT GCT G-3′), (5′-CCG TAG AGG CTC AGG ATA GCC AC-3′) and (5′-GAC GAG TTC TTC TGA GGG GAT CGA TC-3′) which distinguished a 379 bp fragment in the WT from a 605 bp fragment in samples where *S1pr4* was deleted.

### 4.2. Mast Cell Cultures

Mouse bone marrow-derived mast cells (BMMC) were differentiated from the marrow of tibias and femurs of *S1pr4^+/+^* and *S1pr4^−/−^* littermate mice and cultured for at least 6 weeks in RPMI 1640 supplemented with 10% FBS, 1M HEPES, 100 U/mL penicillin, 100 μg/mL streptomycin, 4 mM l-glutamine, 1 mM sodium pyruvate, 50 μM 2-meraptoethanol, 20 ng/mL IL-3, 20 ng/mL stem cell factor (SCF) and non-essential amino acids, as described [22]. Under these conditions, the proportion of mast cells in culture increases overtime and by 4–6 weeks >98% of the cells are mast cells. Mast cell expand particularly after 20 days in culture, when already >85% of the cells are mast cells. The purity of mast cells in the cultures was monitored by assessing the percentage of cells expressing the receptor for SCF, CD117 (Kit) and the IgE receptor, FcεRI, by flow cytometry. Functional studies were conducted on cultures containing >95% double-positive mast cells as described [62]. The total number of mast cells was calculated as: (Absolute total cell count in the culture X percentage of mast cells (FcεRI^+^/CD117^+^))/100 for each time point. 

Peritoneal mast cells (PDMC) obtained from the peritoneal lavage of these mice were expanded in culture for 2 to 3 weeks in the same culture media as BMMC [22,63,64]. 

### 4.3. Degranulation Assays

Degranulation was assessed by a colorimetric detection of the granule marker, β-hexosaminidase, as described [65]. Briefly, mast cells were sensitized with 100 ng/mL anti-DNP IgE (clone H1-DNPε-26.82) [66] overnight in cytokine-free medium. Cells (3 × 10^4^ PDMC or 5 × 10^4^ BMMC) were plated in 96-well 340 µL V-bottom polypropylene (Corning, New York, NY, USA) plates in 100 µL of HEPES buffer (10 mM HEPES, 137 mM NaCl, 2.7 mM KCl, 0.4 mM Na_2_HPO_4_·7H_2_O, 5.6 mM Glucose, 1.8 mM CaCl_2_·2H_2_O, 1.3 mM MgSO_4_·7H_2_O). Cells were stimulated for 30 min with the indicated concentrations of 2,4-Dinitrophenyl-Human Serum Albumin (DNP-HSA) (Sigma, St. Louis, MO, USA), compound 48/80 trihydrochloride (Abcam, Cambridge, MA, USA) and/or recombinant mouse mature IL-33 (Ser 109 through Ile 266, Accession #AK075849) (eBioscience, Waltham, MA, USA). Cells were then centrifuged and supernatants separated from the cell pellets. Fifty µL of the supernatants and cell pellets lysed in 200 µL of 0.1% Triton X were transferred to 96-well plates to determine β-hexosaminidase activity. Degranulation was expressed as the percentage of β-hexosaminidase activity released into the media compared to total cellular β-hexosaminidase activity.

### 4.4. Flow Cytometry

Cells were resuspended at 10^7^ cells/mL in PBS + aqua live/dead stain (Thermo Fisher, Waltham, MA, USA) according to manufacturer’s instructions. Cells were then washed and resuspended in FACS buffer (PBS + 2% FCS + 0.05% sodium azide) and a total of 10^6^ cells (10^7^ cells/mL) were incubated with anti-CD16/CD32 (clone 2.4G2—BD Pharmingen, San Jose, CA, USA), then stained with an optimal amount of antibody conjugate; anti-CD117-APC (1:500) (clone ACK2—eBioscience), anti-IgE-FITC (1:100) (clone R35-72—BD Bioscience), anti-FcεRI-FITC (1:200) or -PE (1:1000) (clone MAR-1—eBioscience). Data acquisition was performed on a LSR II flow cytometer (BD Biosciences, Sparks, MD, USA) and analyzed using FlowJo software (Tree Star, Ashland, OR, USA).

### 4.5. Measurement of Cytokine Release

Mast cells (10^6^/mL) were sensitized with anti-DNP IgE (100 ng/mL) overnight in culture media. Cells were washed ×3 and 10^6^ cells plated in triplicate to 48-well plates in a volume of 1 mL of cytokine-free RPMI/well and stimulated with the indicated concentrations of DNP-HSA. After 4 h, supernatants were collected and IL-6 and TNF-α secretion was measured by ELISA (R & D systems, Minneapolis, MN, USA) as described [62].

### 4.6. RT-PCR and Gene Expression Analysis

Mast cells were sensitized and challenged with 25 ng/mL DNP-HSA (or media alone for unstimulated controls) as for cytokine release described above for 2 or 4 h, as indicated. Total RNA from 10^6^ mast cells was isolated using the RNAeasy plus mini-kit (Qiagen, Germantown, MD, USA) according to the manufacturer’s instructions with inclusion of the QIAShredder step. RNA quantity and purity were determined using the NanoDrop ND-2000 (Nanodrop Technologies, Wilmington, DE, USA).

For qPCR analysis, 1 µg of total RNA was reverse-transcribed using the SuperScript III first-strand synthesis system with random hexamer primers (ThermoFisher, Waltham, MA, USA). cDNA and corresponding reactions in samples without reverse transcription were assessed, in triplicate, by real-time PCR using the CFX96 Sequence Detection System (BioRad, Hercules, CA, USA). Gene-specific cDNA was amplified using Taqman gene expression probes (Table S3). The threshold cycle method, i.e., Δ*C_T_*, was used to quantify the relative abundance of each cDNA, using corresponding *Gapdh* (glyceraldehyde-3-phosphate dehydrogenase) levels for normalization [67]. The *C_T_* values in control samples without reverse-transcription did not exceed background levels.

For RT^2^ gene expression array analysis, cDNA was generated from total RNA (0.5 µg) using the RT^2^ First Strand Kit (Qiagen, Germantown, MD, USA) mixed with RT^2^ SYBR Green qPCR Master Mix (Qiagen, Germantown, MD, USA) and aliquoted onto mouse cytokine & chemokine PCR-array plates (PAMM-150ZD-12—Qiagen). All steps were done according to the manufacturer’s protocol for the BioRad CFX96 Sequence Detection System. Data normalization was based on correcting all *C*_t_ values for the average *C*_t_ values of several consistently expressed housekeeping genes (HKGs) present on the array. Data was analyzed online using the manufacturer’s website (http://pcrdataanalysis.sabiosciences.com/pcr/arrayanalysis.php).

For droplet digital PCR (ddPCR) analysis, cDNA was generated from total RNA (160 ng) using the iScript Advanced cDNA synthesis kit for RT–qPCR (BioRad, Hercules, CA, USA). The PrimePCR ddPCR gene expression probe assay was carried out according to the manufacturer’s suggested protocol using *Gapdh* for high expressing transcripts or *Hprt* for low-expressing transcripts as reference probes (Table S2). Droplets for each sample were generated using a BioRad Dropplet generator and transferred to a 96-well PCR plate. PCR reactions in the droplets were performed utilizing a 2-step thermocycling protocol [95 °C × 10 min; 40 cycles × [(94 °C × 30 s, 60 °C × 60 s); 98 °C × 10 min, ramp rate set at 2.5 °C/s]] in a BioRad C1000 Touch thermocyler and the number of transcripts in each well determined using a QX100 Droplet Reader. Data was analyzed using QuantaSoft analysis software (BioRad, Hercules, CA, USA).

### 4.7. Chemotaxis

*S1pr4*^+/+^ and *S1pr4*^−/−^ BMMC were incubated overnight in serum-free, cytokine-free RPMI supplemented with 0.04% fatty acid-free BSA (FAF-RPMI) with or without 100 ng/mL anti-DNP IgE (clone H1-DNPɛ-26.82) [66]. Cells were then washed twice and suspended in 100μl FAF-RPMI in the top wells of a 5 μm pore size Transwell plate (Costar, Tewksbury MA, USA) in triplicate for 30 min, with 600 μL FAF-RPMI in the lower chamber. The cells in the upper wells were then transferred to a test well containing 600 μL FAF-RPMI supplemented with the indicated concentrations of DNP-HSA, 10 ng/mL SCF or FAF-RPMI only as a negative control. Wells were incubated for 4 h at 37 °C, 5% CO_2_. Cells and media in the lower chamber were then spun down, resuspended with 15μl PBS and counted using a LUNA-FL cell counter (Logos Biosystems, Annandale, VA, USA). Total cells counts were calculated and averaged from 3 technical replicates for each experimental condition.

### 4.8. Passive Systemic Anaphylaxis

*S1pr4^+/+^* or *S1pr4^−/−^* mice were sensitized (i.v.) with 3 μg DNP-specific IgE (clone H1-DNPɛ-26.82) [66] (0.2 mL volume) and challenged 24 h later by i.v. injection of 9 μg purified monoclonal rat anti-mouse IgE (BD Biosciences). IPTT Implantable electronic transponders (BioMedic Data Systems, Inc.—Seaford, DE, USA) were inserted under the dorsal skin fold immediately prior to the systemic administration of IgE. All injections were conducted on anesthetized mice (2% isoflurane, 98% oxygen mix for 2 to 3 min) in a closed chamber. Basal body temperature was determined prior to induction of anaphylaxis with anti-IgE and changes in temperature were measured using a DAS-8007 wireless reader system (BioMedic Data Systems, Inc., Seaford, DE, USA) at the indicated time intervals for a total period of 1 h.

### 4.9. Statistical Analysis

Statistical analysis comparing two grups was performed using a two-tailed unpaired *t*-test or two-way ANOVA if indicated (prism version 7.0; GraphPad Software, Inc., San Diego, CA, USA). Differences were considered significant when *p* < 0.05.

## Supplementary Materials

Supplementary materials can be found at http://www.mdpi.com/1422-0067/19/5/1279/s1.

## Abbreviations

GPCR	G Protein-coupled receptor
S1P	Sphingosine-1-phosphate
S1P_1-5_	Sphingosine-1-phosphate receptors 1-5
S1PR	Sphingosine-1-phosphate receptor
FcεRI	High affinity IgE receptor, Fc Epsilon receptor I
Ag	Antigen
SCF	Stem cell factor
BMMC	Bone marrow-derived mast cells
PDMC	Peritoneum-derived mast cells
WT	Wild type
qPCR	Quantitative real-time PCR
FAF BSA	Fatty acid-free bovine serum albumin
ddPCR	Droplet digital PCR
PSA	Passive systemic anaphylaxis
mAb	Monoclonal antibody
