# Supplementary material for: S1P4 Regulates Passive Systemic Anaphylaxis in Mice but Is Dispensable for Canonical IgE-Mediated Responses in Mast Cells
Source: Int J Mol Sci. 2018 Apr 25;19(5):1279. doi: 10.3390/ijms19051279 (PMC5983835; doi:10.3390/ijms19051279)
Supplement: Supplementary file 1 [file ijms-19-01279-s001.pdf]

Supplementary

S1P4 Regulates Passive Systemic Anaphylaxis in Mice but Is Dispensable for Canonical IgE-Mediated Responses in Mast Cells

Joseph M. Kulinski [1], Richard L. Proia [2], Elisabeth M. Larson [1], Dean D. Metcalfe [1] and Ana Olivera [1,*]

1 Mast Cell Biology Section, Laboratory of Allergic Diseases, National Institute of Allergy and Infectious Diseases (NIAID), National Institutes of Health, Bethesda, MD 20892, USA; Joseph.kulinski@nih.gov (J.M.K.); eml244@cornell.edu (E.L.); dmetcalfe@niaid.nih.gov (D.D.M.)

2 Genetics of Development and Disease Branch, National Institute of Diabetes and Digestive and Kidney Diseases (NIDDK), National Institutes of Health, Bethesda, MD 20892, USA; richardp@intra.niddk.nih.gov

* Correspondence: Ana.Olivera@nih.gov

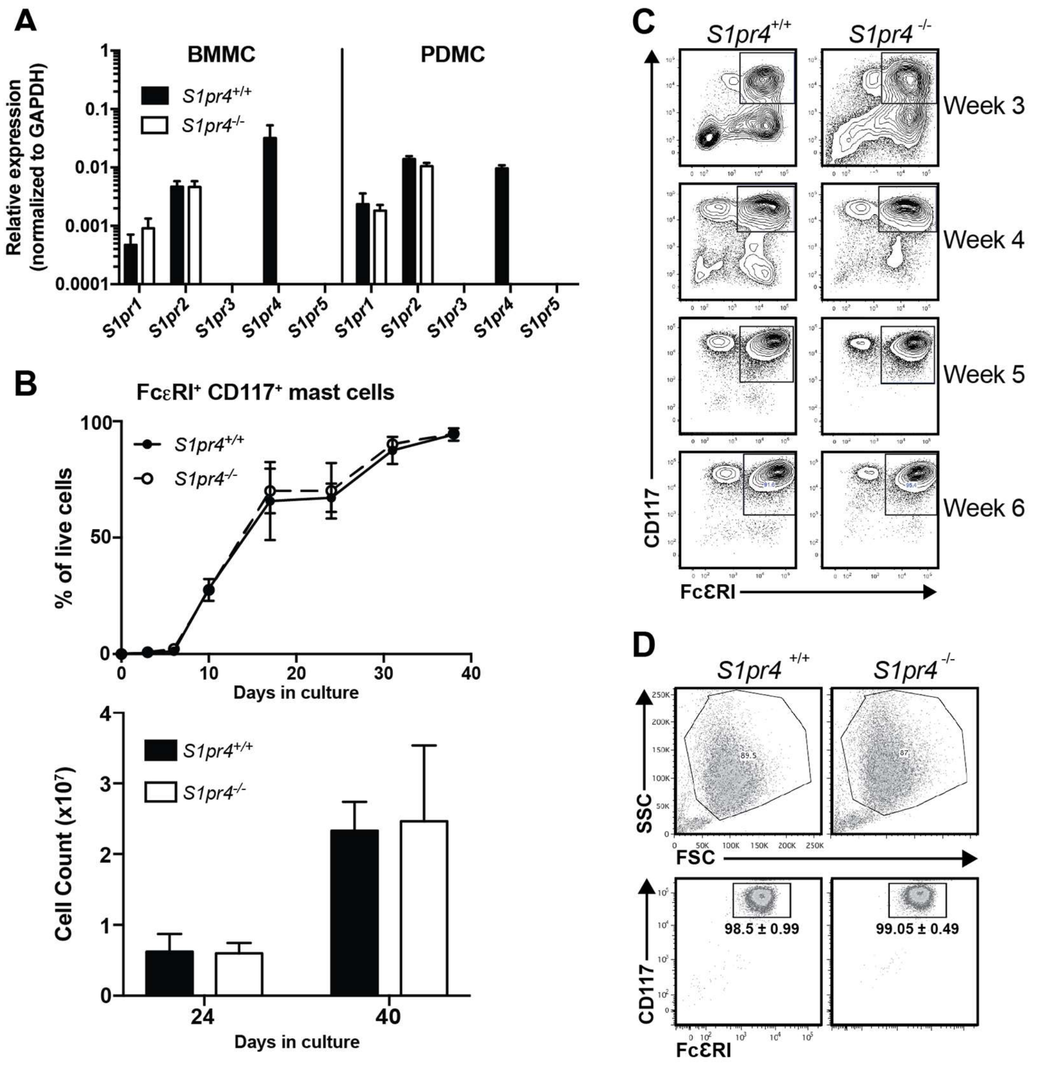

Figure S1. Growth and differentiation of murine mast cells in the absence of S1pr4 expression **A)** Quantitative PCR data showing the relative expression of S1P receptor message normalized to GAPDH in cultured BMMC (left) and PDMC (right) from *S1pr4*$^{+/+}$ (solid bars) and *S1pr4*$^{-/-}$ mice (open bars). In BMMC, bars represent the mean ± SE of data pooled from 7 independent

WT and 4 *S1pr4*$^{-/-}$ cultures and in PDMC, 2 independent WT and 4 *S1pr4*$^{-/-}$ cultures . **B,C)** Frequency (Panel B, top) and absolute number (Panel B, bottom) of mast cells (FcεRI^{+}/CD117^{+}) over time in culture. Bone marrow cells harvested from *S1pr4*$^{+/+}$ (black) and *S1pr4*$^{-/-}$ mice (grey) were grown in the presence of SCF and IL-3 for 7 weeks. Non-adherent cells were counted and analyzed by flow cytometry. The gating strategy used to identify mast cells is represented in panel C. Data represent the mean ± SE of at least 3 independent cultures. **D)** Representative flow cytometry plots showing the forward and side-scatter profile (top) and mast cell marker surface expression profile (bottom) profiles of non-adherent cells expanded from peritoneal exudates of *S1pr4*$^{+/+}$ (left) and *S1pr4*$^{-/-}$ mice (right). The frequency of FcεRI^{+}/CD117^{+} represents mean ± SE at least 3 independent experiments.

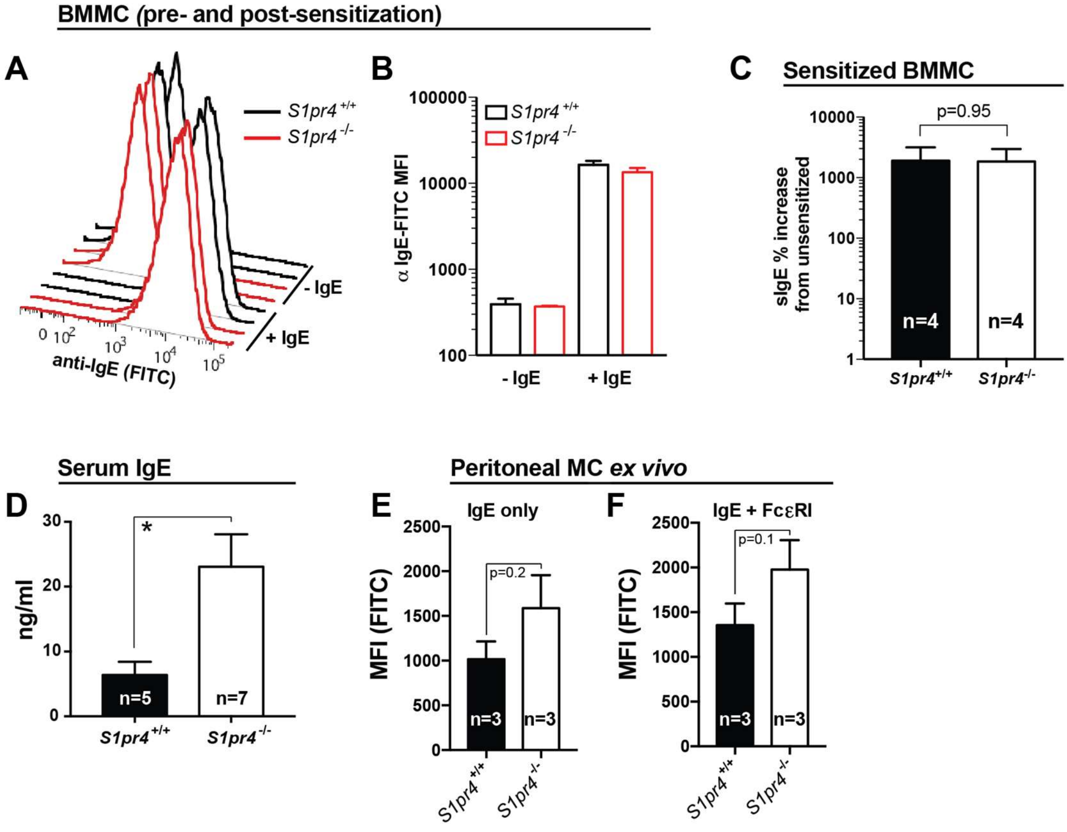

Figure S2. IgE bound by mast cells and in circulation. **A-C)** Surface bound IgE was measured by FACS on cultured BMMC before (control, -IgE) and after sensitization with 100 ng/ml anti-DNP IgE overnight (+IgE) in cytokine-free media. **A)** graphical histogram of raw FACS data from a single representative experiment comparing 2 WT and 2 *S1pr4*$^{-/-}$ BMMC cultures grown and analyzed concurrently. **B)** Average anti-IgE FITC median fluorescence intensity (MFI) of the 2 independent cultures for each genotype shown in A. Bars represent mean MFI ± SD. **C)** Percent increase in MFI in sensitized compared to control WT or *S1pr4*$^{-/-}$ BMMC. These data were pooled from 4 independent cultures of each genotype and represent mean ± SE. **D)** Mean ± SE serum IgE levels measured from 6 to 8 week old *S1pr4*$^{+/+}$ and *S1pr4*$^{-/-}$ mice by ELISA. **E,F)** Peritoneal exudate cells (PEC) from 3 *S1pr4*$^{+/+}$ and 3 *S1pr4*$^{-/-}$ mice were stained directly *ex vivo* with anti-IgE-FITC and anti-CD117-APC (E) or anti-IgE-FITC, anti-FcεRI-FITC and anti-CD117-APC (F) for FACS analysis. Pooled data shows the mean ± SE MFI of anti-IgE FITC on CD117^{+} pre-gated peritoneal mast cells and is representative of 2 independent experiments.***p=<0.05**

Table S1. Normalized relative mRNA expression values for select genes from RT^2 cytokine array analysis.

	Relative Expression[1] (unstimulated, n=2)		Relative Expression[1] (IgE+Ag, n=2)		Average fold change[2] (IgE+Ag, n=3)
**Gene**	***S1pr4*** $^{+/+}$	***S1pr4*** $^{-/-}$	***S1pr4*** $^{+/+}$	***S1pr4*** $^{-/-}$	***S1pr4*** $^{-/-}$ / ***S1pr4*** $^{+/+}$
Ccl1	0.0087	0.0093	0.4406	0.8815	1.7
Ccl3	0.1407	0.1448	0.4951	0.7972	1.4
Ccl4	0.0413	0.0556	0.2091	0.3385	1.2
Ccl7	1.0121	0.8870	1.4941	1.8698	1.3
Ccl12	0.0007	0.0007	0.0034	0.0072	5.8
Ccl17	0.0279	0.0527	0.0363	0.0263	1.5
Cxcl3	0.0002	0.0002	0.0002	0.0003	1.2
Ccl22	0.0002	0.0002	0.0001	0.0002	6.7
Ccl24	0.0007	0.0013	0.0003	0.0005	5.0
Il1a	0.0037	0.0069	0.0140	0.0234	1.6
Il2	0.0002	0.0002	0.0008	0.0017	5.6
Il5	0.0002	0.0002	0.0063	0.0093	4.1
Il9	0.0004	0.0004	0.0007	0.0018	2.4
Il11	0.0002	0.0002	0.0002	0.0003	1.1
Il13	0.0513	0.0775	0.4972	0.8664	1.5
Csf2	0.0077	0.0036	0.0253	0.0476	1.4
Ifng	0.0002	0.0002	0.0004	0.0004	1.1

[1]Values represent normalized relative mRNA expression of select genes by bone marrow-derived mast cells (BMMC) as measured by RT^2 quantitative PCR array analysis.

[2]Values represent the average fold difference between relative expression in Ag-stimulated *S1pr4* $^{-/-}$ BMMC and Ag-stimulated *S1pr4* $^{+/+}$ wildtype conrol cells.

n indicates the number of independent cultures represented in each value.

Table S2. Probes used for gene expression analysis.

Gene	Probe	Application	Manufacturer
S1pr1	Mm00514644_m1	qPCR	Thermo Fisher
S1pr2	Mm01177794_m1	qPCR	Thermo Fisher
S1pr3	Mm02620181_s1	qPCR	Thermo Fisher
S1pr4	Mm00468695_s1	qPCR	Thermo Fisher
S1pr5	Mm02620565_s1	qPCR	Thermo Fisher
Il6	Mm00446190_m1	qPCR	Thermo Fisher
Tnfa	Mm00443258_m1	qPCR	Thermo Fisher
Gapdh	Mm99999915_g1	qPCR	Thermo Fisher
Ccl1	dMmuCPE5090242	ddPCR	BioRad
Ccl12	dMmuCPE5107958	ddPCR	BioRad
Ccl17	dMmuCPE5088392	ddPCR	BioRad
Ccl22	dMmuCPE5116290	ddPCR	BioRad
Ccl24	dMmuCPE5123514	ddPCR	BioRad
Il2	dMmuCPE5107882	ddPCR	BioRad
Il5	dMmuCPE5110012	ddPCR	BioRad
Il6	dMmuCPE5095532	ddPCR	BioRad
Il9	dMmuCPE5112064	ddPCR	BioRad
Gapdh	dMmuCPE5195283	ddPCR	BioRad
Hprt	dMmuCPE5095492	ddPCR	BioRad
